# Draft Genome Sequence of *Salmonella enterica* subsp. *enterica* Serovar Bardo Strain CRJJGF_00099 (Phylum *Gammaproteobacteria*)

**DOI:** 10.1128/genomeA.00964-16

**Published:** 2016-09-15

**Authors:** Sushim K. Gupta, Elizabeth A. McMillan, Charlene R. Jackson, Prerak T. Desai, Steffen Porwollik, Michael McClelland, Lari M. Hiott, Shaheen B. Humayoun, Jonathan G. Frye

**Affiliations:** a Bacterial Epidemiology and Antimicrobial Resistance Research Unit, USDA-ARS, Athens, Georgia, USA; b Department of Microbiology, University of Georgia, Athens, Georgia, USA; c Department of Microbiology and Molecular Genetics, University of California, Irvine, Irvine, California, USA

## Abstract

Here, we report a 4.87-Mbp draft genome sequence of the multidrug-resistant (MDR) *Salmonella enterica* subsp. *enterica* serovar Bardo strain CRJJGF_00099, isolated from dairy cattle in 2005.

## GENOME ANNOUNCEMENT

*Salmonella enterica* subsp. *enterica* serovar Bardo is a rarely reported serotype across the globe; however, *S.* Bardo has been reported from chicken carcasses in Portugal (1), pet reptiles in Japan (2), and oysters in the United States (3). *S.* Bardo has also been previously isolated from cattle in the United States (4), and an *S.* Bardo outbreak was reported in Switzerland in 2011 (5). Here, we announce the draft genome sequence of a multidrug-resistant (MDR) *S.* Bardo strain isolated from dairy cattle in 2005.

Standard microbiology techniques were applied to isolate *Salmonella* strains from food animals. Using pulsed-field gel electrophoresis (PFGE), as described by PulseNet (6), the isolate was assigned PFGE pattern TEGX01.0001.ARS. The isolates were serotyped using SMART (7), and sequencing reads were used to determine an antigenic formula to predict the serotype using SeqSero (8), which predicted the antigenic formula of 8:e,h:1,2 designated *S.* Bardo. As *S.* Bardo shares the same antigenic profile with *Salmonella enterica* subsp. *enterica* serovar Newport, *S.* Bardo was confirmed with the help of the PFGE profile, SMART typing pattern, and antigenic formula. Susceptibility testing for the strain was performed using broth microdilution plates for the Sensititre semiautomated antimicrobial susceptibility system (Trek Diagnostic Systems, Inc., Westlake, OH). The results were interpreted according to Clinical and Laboratory Standards Institute (CLSI) guidelines (9).

Genomic DNA was isolated from an overnight culture using the GenElute bacterial genomic DNA kit (Sigma-Aldrich, St. Louis, MO). DNA libraries were constructed using Nextera-XT DNA preparation kit, and paired-end sequencing was performed on the Illumina HiSeq 2500 (Illumina, Inc., San Diego, CA) using a 500-cycle MiSeq reagent kit. A total of 5,050,994 reads were generated. The reads were *de novo* assembled using Velvet (10), which assembled to 190 contigs ≥200 bp, with 96-fold average coverage. The combined length of the contigs was 4,875,034 bases, with a G+C content of 52.17% and an *N*_50_ value of 60.1 kb. The contigs were ordered with Mauve (11) using the *Salmonella* LT2 genome sequences as a reference, and coding sequences were predicted with Prodigal (12). A total of 4,598 coding sequences (≥50 amino acids) were predicted within the genome. Signal peptide, clustered regularly interspaced short palindromic repeat (CRISPR) regions, and prophages were predicted using SignalP (13), CRISPRFinder (14), and PHAST (15), respectively. We identified signal peptides in 449 coding sequences (CDSs), two CRISPR loci, and five intact phage, including Salmon_SP_004 (accession no. NC021774), Gifsy2 (accession no. NC010393), Salmon_SEN34 (accession no. NC028699), and two Gifsy_1 (accession no. NC010392) phage in the analyzed contigs. The MDR isolate was resistant to streptomycin, ampicillin, cefoxitin, ceftiofur, chloramphenicol, sulfisoxazole, and tetracycline, and we identified corresponding resistance genes [*strA*, *strB*, *cmy*-94, *floR*, *sulII*, and *tet*(A)] and a cryptic aminoglycoside resistance gene, *aac6*-Iy, through ARG-ANNOT (16). The data generated from the draft genomes can provide useful information about the genomic variations in this rarely reported serovar.

### Accession number(s).

The genome sequence of *Salmonella enterica* subsp. *enterica* serovar Bardo strain CRJJGF_00099 has been deposited in the GenBank database (NCBI) under the accession no. JQWD00000000. This paper describes the first version of the genome.
